# Exploring the Association Between Breast Cancer Perception and Health-Promoting Lifestyle Behaviours Among Women: A Cross-Sectional Study in Saudi Arabia

**DOI:** 10.3390/healthcare14213820

**Published:** 2026-11-09

**Authors:** Lizy Sonia Benjamin, Sumathi Robert Shanmugam, Asma Saad Habbash, Iman Awad Siddig Mohammed, Sahar Rafdan Alshahrani, Mona Alsherim, Maysa M. Mohsen, Mawahib Mohammed Salih Ebrahim, Razan Suliman Al Humayed, Vidhya Thirunavukkarasu, Nagwa Ibrahim Elfeshawy

**Affiliations:** 1 Department of Adult and Advanced Care Nursing, King Khalid University, Abha 61421, Saudi Arabia; ljamin@kku.edu.sa (L.S.B.); malshreem@kku.edu.sa (M.A.); mrajb@kku.edu.sa (M.M.M.); 2 Department of Maternity and Pediatric Nursing, College of Nursing, Princess Nourah Bint Abdulrahman University, Riyadh 11671, Saudi Arabia; 3 Department of Family and Community Medicine, College of Medicine, King Khalid University, Abha 61421, Saudi Arabia; ahabbash@kku.edu.sa (A.S.H.); raalhumaid@kku.edu.sa (R.S.A.H.); 4 Department of Community Health Nursing, College of Nursing, King Khalid University, Abha 61421, Saudi Arabia; immohammed@kku.edu.sa (I.A.S.M.); maebrahim@kku.edu.sa (M.M.S.E.); 5 Department of Administration and Education, College of Nursing, King Khalid University, Abha 61421, Saudi Arabia; srsafar@kku.edu.sa; 6 Department of Nursing, College of Applied Medical Sciences, King Faisal University, Al Ahsa 36362, Saudi Arabia; vmudaliyar@kfu.edu.sa; 7 Maternal and Pediatric Nursing Department, King Khalid University, Abha 61421, Saudi Arabia; nelfeshawy@kku.edu.sa

**Keywords:** breast cancer, breast cancer perception, health-promoting lifestyle, health belief model, mammography, cancer screening, Saudi Arabia

## Abstract

Aim: This study aimed to describe breast cancer perceptions, health-promoting lifestyle behaviours, and lifetime breast examination and mammography practices and to examine associations between breast cancer perception subscales and health-promoting lifestyle behaviours among women in Abha, Saudi Arabia. Methods: This cross-sectional online survey included 1002 Saudi women aged ≥ 18 years living in Abha, recruited by convenience sampling through social media and instant messaging. Arabic-language versions of the Breast Cancer Perception Scale (BCPS) and HPLP-II were self-administered. Breast self-examination (BSE), clinical breast examination (CBE), and mammography were assessed as lifetime ever/never items. Multiple linear regression examined associations of sociodemographic variables and BCPS subscales with HPLP-II scores. Results: Perceived knowledge (3.64 ± 1.05), treatment belief (3.43 ± 0.77), fear (3.25 ± 1.30), and perceived risk (3.40 ± 0.89) had higher mean scores than perceived need for health checks (2.78 ± 1.23) and stigma (2.89 ± 1.14). The HPLP-II mean was 2.63 ± 0.46 (spiritual growth highest, 2.96 ± 0.50; physical activity lowest, 2.23 ± 0.60). Lifetime self-reported prevalence was 61.1% for BSE (95% CI 58.1 to 64.1), 22.5% for CBE (95% CI 19.9 to 25.0), and 12.8% for mammography (95% CI 10.7 to 14.8). Among women aged ≥ 40 years, 68 of 225 reported lifetime mammography (30.2%; 95% CI 24.2 to 36.2). In a mutually adjusted model of BCPS subscales, higher HPLP-II scores were associated with perceived knowledge (unstandardized B = 0.059, 95% CI 0.032 to 0.087, *p* < 0.001), treatment belief (B = 0.120, 95% CI 0.078 to 0.163, *p* < 0.001), and stigma (B = 0.058, 95% CI 0.023 to 0.093, *p* = 0.001), and lower scores were associated with perceived need for health checks (B = −0.259, 95% CI −0.293 to −0.225, *p* < 0.001), perceived risk (B = −0.130, 95% CI −0.176 to −0.083, *p* < 0.001), and fear (B = −0.028, 95% CI −0.054 to −0.003, *p* = 0.030). These associations were not adjusted for sociodemographic characteristics and should be interpreted cautiously. Conclusions: In this online convenience sample, lifetime mammography and CBE were less commonly reported than BSE, and physical activity was the lowest-scoring lifestyle domain. These findings do not show whether awareness or perceptions influence screening participation because screening was not modelled against these constructs. Longitudinal studies with age- and indication-specific screening measures are needed to clarify these relationships.

## 1. Introduction

Worldwide, breast cancer is the most commonly diagnosed malignancy and the leading cause of cancer death among women [1]. According to GLOBOCAN 2022, approximately 2.31 million new breast cancer cases and 670,000 breast cancer deaths occurred globally, accounting for nearly one in four female cancer cases and one in six female cancer deaths [1]. Incidence rates were considerably higher in transitioned than transitioning countries (54.1 versus 30.8 per 100,000), whereas mortality rates were lower (11.3 versus 15.3 per 100,000) [1]. Rapid increases in breast cancer incidence and mortality have also been reported in transitioning countries in South America, Africa, and Asia [1].

Breast cancer is the most commonly diagnosed cancer among women in Saudi Arabia [2]. An analysis of Saudi Cancer Registry data found that the number of cases among Saudi women rose by 351.9%, from 545 in 2001 to 2463 in 2017, while the age-standardized incidence rate increased by 151.7%, from 11.8 to 29.7 per 100,000. By 2017, breast cancer represented 30.9% of all cancers diagnosed in Saudi women [2].

Screening mammography can reduce breast cancer mortality, although recommendations vary by age and guideline. The U.S. Preventive Services Task Force recommends biennial screening mammography for women aged 40 to 74 years [3]. The Saudi Ministry of Health also offers free breast cancer screening through primary care centres and dedicated programmes [4]. However, reported screening use remains low. Studies from Riyadh and the Northern Borders region reported that 41.6% and 33.5% of women, respectively, had ever performed breast self-examination (BSE) [5,6]. In the Aseer region, 6.37% reported always performing BSE [7]. Reported mammography use has varied from 19.5% to approximately 40% across studies in Arar, Aseer, and a separate Saudi sample [6,7,8]. Despite free service provision, a 2024 systematic review and meta-analysis documented low use of BSE, clinical breast examination, and mammography in Saudi Arabia and identified fear of diagnosis, embarrassment, and lack of awareness as key barriers [9].

The gap between awareness and reported practice suggests that knowledge alone may not ensure participation [5,6]. Breast cancer perceptions, including perceived risk, fear, stigma, and treatment beliefs, may help explain screening behaviour, although this study did not examine screening participation as an outcome [10].

Alongside early detection, modifiable lifestyle factors are relevant to breast cancer prevention. The World Cancer Research Fund/American Institute for Cancer Research reports strong evidence that being physically active decreases the risk of postmenopausal breast cancer, whereas greater body fatness throughout adulthood and adult weight gain increase that risk [11]. Previous Saudi studies using the Health-Promoting Lifestyle Profile II (HPLP-II) have reported spiritual growth as the highest-scoring subscale and physical activity as the lowest [12,13]. Examining how breast cancer perceptions relate to these behaviours may help identify areas for prospective prevention research.

Breast cancer perception is a multidimensional concept comprising perceived knowledge, treatment beliefs, perceived need for health checks, stigma, fear, and perceived risk within the Health Belief Model [10]. Evidence examining both breast cancer perception and health-promoting lifestyle behaviours in the Aseer region is limited, and previous research there identified a marked gap between BSE knowledge and practice [7]. This study therefore aimed to describe breast cancer perceptions, health-promoting lifestyle behaviours, and lifetime examination and mammography practices among women living in Abha, Saudi Arabia, and to examine associations between BCPS subscales and HPLP-II scores. We expected perceived knowledge and treatment belief to be positively associated with health-promoting lifestyle scores. Associations involving perceived need for health checks, stigma, fear, and perceived risk were treated as exploratory because the Health Belief Model does not specify a single unambiguous direction for these constructs in relation to general lifestyle behaviour. Lifestyle was modelled as the outcome for analytical clarity, without implying temporal direction.

## 2. Materials and Methods

### 2.1. Study Design and Setting

A cross-sectional descriptive design was employed. The study was conducted in Abha, the capital city of the Aseer Region in southwestern Saudi Arabia, between December 2025 and May 2026.

### 2.2. Population, Sampling, and Data Collection

Participants were recruited by convenience sampling through social media and instant messaging using a Google Forms questionnaire. Google account login verification, a one-response-per-account setting, and browser/account controls were used to reduce duplicate submissions. This strategy may preferentially include women with internet access, greater digital engagement, or an interest in breast cancer. The final sample comprised 1002 self-identified Saudi women who reported residing in Abha, were aged 18 years or older, had no previous breast cancer diagnosis, could read and write Arabic, and consented to participate. Eligibility, nationality, and residence were self-reported. Because the survey was distributed through open online links, the numbers of views and invitations were unavailable, and a participation rate could not be calculated.

The minimum sample size was initially calculated using the single-proportion formula n_0_ = Z^2^p (1 − p)/d^2^ [14]. Assuming *p* = 0.50, a 95% confidence level (Z = 1.96), and d = 0.031 gives n_0_ = 999.38; rounding upward yielded a minimum target of 1000 women. This calculation concerned precision for a single proportion under simple random-sampling assumptions and was not a power calculation for the regression models. A separate fixed-model multiple-regression calculation with 14 predictors, f^2^ = 0.05, α = 0.05, and 90% power indicated a minimum of 473 participants [15]. As supplementary benchmarks, Green’s rules give N ≥ 50 + 8 m = 162 for testing the multiple correlation and N ≥ 104 + m = 118 for testing partial correlations when m = 14, although these rules assume a medium-size relationship and do not replace an effect-size-based calculation [16]. The achieved sample of 1002 exceeded these requirements and provided power > 0.99 for the specified f^2^ = 0.05 calculation; this does not by itself guarantee coefficient stability or satisfaction of regression assumptions. The questionnaire was administered online and took approximately 15 min to complete it. Suspected duplicate submissions were identified using platform duplicate/identifier indicators together with matching timestamps and demographic profiles.

### 2.3. Data Collection Instruments

Data were collected using a self-administered questionnaire comprising two instruments alongside sociodemographic and screening-practice items. Sociodemographic variables were age (continuous), marital status (married; unmarried [single/divorced/widowed]), education (primary; high schooling [completion of Grades 10–12 in the general curriculum]; secondary schooling [vocational diploma or technical institute post-secondary certification]; and university [bachelor’s/postgraduate]), occupation (not working [students/homemakers/unemployed], public official [government/education/healthcare], and self-employed), and family history of breast cancer (yes/no). 

#### 2.3.1. Arabic Translation and Adaptation

Both instruments were translated into Arabic using a forward and backward translation process based on international guidance [17]. Two independent bilingual translators produced Arabic translations and agreed on a single version. A third translator, a native English speaker working independently, translated this version back into English. The primary investigators and original scale authors compared the back-translated version with the originals to assess conceptual, semantic, and item-level equivalence. This process supports linguistic equivalence; reliability and other psychometric properties were assessed separately.

#### 2.3.2. Health-Promoting Lifestyle Profile II (HPLP-II)

The HPLP-II, developed by Walker and Hill-Polerecky [18] measures health-promoting behaviours through 52 items divided into six subscales: health responsibility (9 items), spiritual growth (9 items), physical activity (8 items), interpersonal relations (9 items), nutrition (9 items), and stress management (8 items). Each item is rated on a 4-point Likert-type scale (1 = never, 2 = occasionally, 3 = frequently, 4 = routinely). Subscale and overall scores were calculated as unweighted arithmetic means across the exact item counts for complete cases, without imputation, and analysed as continuous measures; higher scores indicate more frequent health-promoting behaviour. Scores were not categorized as moderate or positive. The original Arabic validation concerned the earlier HPLP and does not by itself establish the validity of the complete 52-item HPLP-II in a comparable Saudi population. In the present study, Cronbach’s alpha was 0.89 for the total scale, 0.85 for health responsibility, 0.88 for physical activity, 0.79 for nutrition, 0.89 for spiritual growth, 0.82 for interpersonal relations, and 0.84 for stress management.

#### 2.3.3. Breast Cancer Perception Scale (BCPS)

The BCPS, developed by Taylan et al. [10] based on the Health Belief Model, contains 24 items across six subscales: perceived knowledge, perceived treatment belief, perceived need for a health check, perceived stigma, perceived fear, and perceived risk. The original item-to-subscale mapping is as follows: perceived knowledge, items 1–4 (4 items); perceived treatment belief, items 5–9 (5 items); perceived need for a health check, items 10–13 (4 items); perceived stigma, items 14–17 (4 items); perceived fear, items 18–21 (4 items); and perceived risk, items 22–24 (3 items) [10]. Responses are scored from 1 to 5. Items 7, 9, 10, 11, 12, and 13 were reverse-coded before calculating subscale means. Higher subscale scores indicate greater levels of the respective construct, not uniformly more favourable perception. The original scale reported subscale Cronbach’s alpha coefficients ranging from 0.815 to 0.950 [10]. In the present sample, Cronbach’s alpha coefficients were 0.88 for perceived knowledge, 0.83 for treatment belief, 0.86 for need for a health check, 0.81 for stigma, 0.91 for fear, and 0.84 for perceived risk. Because the six domains are conceptually distinct and do not share a uniform direction, the previously used sum of subscale means was removed, and no composite BCPS score was analysed.

#### 2.3.4. Examination and Mammography History

Three study-specific questions were used: (1) “Have you ever performed breast self-examination?”; (2) “Have you ever undergone clinical breast examination by a healthcare professional?”; and (3) “Have you ever undergone mammography?” Each item had Yes/No response options. These questions assessed lifetime history and specified no shorter recall interval. They did not establish when the examination occurred or whether CBE or mammography was performed for routine screening, diagnostic investigation, or another indication. BSE was treated as a self-examination practice and not as equivalent to population mammography screening; none of the items was used as a measure of guidelitane-concordant screening coverage.

### 2.4. Statistical Analysis

Data were analysed using IBM SPSS Statistics version 26 (IBM Corp., Armonk, NY, USA). Means, standard deviations, ranges, frequencies, and percentages summarized perception scores, HPLP-II scores, and lifetime examination and mammography history. Ninety-five percent confidence intervals for sample proportions were calculated using a binomial normal approximation and are descriptive; they do not account for convenience sampling or support population-representative inference. Analyses used complete cases without imputation. Coefficients are reported as unstandardized B values with 95% model-based t confidence intervals and two-sided *p*-values. HPLP-II scores were treated as approximately continuous outcomes and BCPS subscale means as approximately continuous predictors. 

An exploratory six-predictor model was developed using only the BCPS subscales, without sociodemographic adjustment. Overall model fit statistics, including R^2^, adjusted R^2^, and the F-statistic, as well as complete regression diagnostics, were not preserved in the primary analysis files and remain unavailable. Consequently, no assumption checks are claimed for this exploratory model, and its findings are interpreted cautiously in comparison with the primary fully adjusted model.A separate fully adjusted model included 14 predictors: age, marital status, high schooling, secondary schooling, university education, public-official status, self-employment, family history, and all six BCPS domains. All predictors were entered simultaneously, without stepwise selection. Categorical reference groups are indicated in the respective tables. No adjustment for multiple comparisons was applied. As a result, the findings should be considered exploratory, and p-values particularly those that are close to 0.005 and 0.05 should be considered nominal.For the primary and fully adjusted models, linearity and homoscedasticity were assessed using standardized residual-versus-predicted plots, residual normality was assessed using Q-Q plots and the Kolmogorov–Smirnov test (p > 0.05) and influential observations were examined using Cook’s distance (0.000 to 0.032). Variance inflation factor (VIF) values ranged from 1.02 to 2.61 in the primary model and from 1.09 to 1.74 in the fully adjusted model. For the sensitivity analysis, the fully adjusted model was refitted after excluding participants with primary education (*n* = 32) and self-employed participants (*n* = 6), leaving 964 cases. Coefficients for marital status, public-official status, and the BCPS domains changed by no more than 4%. Education contrasts using primary education as the reference could not be compared directly after that group was removed.

### 2.5. Ethical Considerations

The study protocol was approved by King Khalid University Institutional Review Board (ECM#2024-3173; approval date: 11 December 2025). The available study records specify recruitment only by month (December 2025 to May 2026) and do not provide the exact first recruitment date. The precise day-level sequence of initial recruitment and IRB approval therefore could not be verified. Electronic informed consent was obtained from all participants before they completed the questionnaire. Participation was voluntary, and no names or direct contact details were requested in the questionnaire.

## 3. Results

### 3.1. Participant Characteristics

Of the 1148 responses accessed, 146 were excluded (42 ineligible, 31 suspected duplicates, and 73 incomplete), leaving 1002 complete cases for analysis. Table 1 summarizes the participant characteristics. The mean age was 32.4 years (SD 9.8; observed range 18–68), and the largest age group was 18–29 years (*n* = 465, 46.4%). Most participants were married (*n* = 584, 58.3%), had university education (*n* = 671, 67.0%), and were public officials (*n* = 568, 56.7%). The primary-education reference category comprised 32 participants (3.2%), and the self-employed subgroup comprised six participants (0.6%); estimates involving these small categories require caution.

### 3.2. Descriptive Characteristics of Perception Scores and Lifestyle Domains

Table 2 summarizes the distribution of the breast cancer perception and HPLP-II subscale scores. Among the perception subscales, perceived knowledge had the highest mean score (3.64, SD 1.05), followed by perceived treatment belief (3.43, SD 0.77) and perceived risk (3.40, SD 0.89). Perceived need for a health check had the lowest mean score (2.78, SD 1.23). For the HPLP-II subscales, spiritual growth had the highest mean score (2.96, SD 0.50), and physical activity had the lowest (2.23, SD 0.60). The mean overall HPLP-II score was 2.63 (SD 0.46, observed range 2.00–4.00). Scores are presented descriptively without assigning moderate or positive categories.

### 3.3. Self-Reported Breast Examination and Mammography History

Table 3 presents lifetime self-reported examination and mammography history overall and by age group. In the full sample, 612 participants reported BSE (61.1%, 95% CI 58.1–64.1), 225 reported CBE (22.5%, 95% CI 19.9–25.0), and 128 reported mammography (12.8%, 95% CI 10.7–14.8). The proportion reporting mammography was 3.9% at ages 18–29 years, 13.5% at ages 30–39 years, 30.4% at ages 40–49 years, and 29.9% at age ≥50 years. Among the 225 women aged ≥40 years, 68 reported lifetime mammography (30.2%, 95% CI 24.2–36.2). These figures describe lifetime history only; they do not establish the clinical indication, examination frequency, or adherence to recommended screening intervals.

### 3.4. Multivariable Linear Regression Examining Sociodemographic Correlates of HPLP-II

In the multivariable linear regression model for HPLP-II score (R^2^ = 0.288, adjusted R^2^ = 0.282, *n* = 1002), as in Table 4, the marital status showed the largest coefficient: married participants scored 0.51 points higher than unmarried participants (B = 0.51, 95% CI 0.44–0.58, *p* < 0.001). Age was inversely associated with HPLP-II score, although the magnitude was small (B = −0.003 per year, 95% CI −0.006 to 0.000, *p* = 0.038). Relative to participants with primary education, those with secondary school education had lower HPLP-II scores (B = −0.53, 95% CI −0.64 to −0.42, *p* < 0.001), and those with university education had a smaller inverse association (B = −0.09, 95% CI −0.17 to −0.01, *p* = 0.031). High school education did not differ significantly from primary education (B = −0.01, *p* = 0.864). This non-monotonic pattern should not be interpreted as evidence that lower education promotes healthier lifestyles.

**Table 4:** Multivariable linear regression examining sociodemographic correlates of HPLP-II score.

Variable	B (Unstandardized)	SE	T	*p*	95% CI
Age (continuous)	−0.0030	0.0014	−2.08	0.038	−0.0058 to −0.0002
Married (ref. Unmarried)	0.5098	0.0365	13.98	<0.001	0.4382 to 0.5814
High Schooling (ref. Primary)	−0.0072	0.0419	−0.17	0.864	−0.0893 to 0.0750
Secondary Schooling (ref. Primary)	−0.5277	0.0553	−9.55	<0.001	−0.6361 to −0.4192
University (ref. Primary)	−0.0869	0.0402	−2.16	0.031	−0.1658 to −0.0080
Public official (ref. Not working)	0.1548	0.0388	3.99	<0.001	0.0787 to 0.2308
Self-employed (ref. Not working)	0.0752	0.1621	0.46	0.643	−0.2429 to 0.3934
Family history: Yes (ref. No)	0.0487	0.0390	1.25	0.212	−0.0278 to 0.1253
Constant	2.4182	0.0688	35.13	<0.001	2.2831 to 2.5533

Note: *n* = 1002; Residual df = 993; R^2^ = 0.288; adjusted R^2^ = 0.282. B denotes unstandardized coefficients. Education and occupation were categorical. The primary-education reference category comprised *n* = 32, and the self-employed category comprised *n* = 6; the sensitivity analysis described in statistical analysis examined the influence of these small categories. VIF range = 1.02–2.61.

Occupation was also associated with the HPLP-II score. Public officials scored higher than participants who were not working (B = 0.15, 95% CI 0.08–0.23, *p* < 0.001). The self-employed group comprised only six participants, and its coefficient was imprecise and not statistically significant (B = 0.08, 95% CI −0.24 to 0.39, *p* = 0.643). Given the small group and imprecise estimate, no substantive interpretation is warranted. Family history of breast cancer was not associated with HPLP-II score (B = 0.05, 95% CI −0.03 to 0.13, *p* = 0.212). Variance inflation factors for all retained predictors ranged from 1.02 to 2.61.

### 3.5. Exploratory Multivariable Linear Regression Examining Associations Between Breast Cancer Perception Subscales and HPLP-II Score

In the Table 5, the exploratory model containing all six perception subscales but no sociodemographic covariates, all six subscales were associated with the HPLP-II score. Perceived treatment belief had the largest positive coefficient (B = 0.12, 95% CI 0.08–0.16, *p* < 0.001), followed by perceived knowledge (B = 0.06, 95% CI 0.03–0.09, *p* < 0.001) and perceived stigma (B = 0.06, 95% CI 0.02–0.09, *p* = 0.001). Perceived need for a health check had the largest inverse coefficient (B = −0.26, 95% CI −0.29 to −0.22, *p* < 0.001), and perceived risk (B = −0.13, 95% CI −0.18 to −0.08, *p* < 0.001) and perceived fear (B = −0.03, 95% CI −0.05 to −0.003, *p* = 0.030) were also inversely associated. Need for health checks had the largest absolute coefficient and fear the smallest. These magnitudes should be interpreted alongside predictor variability, potential confounding, and the exploratory model specification; their practical importance is not established.

**Table 5:** Exploratory multivariable linear regression examining associations between breast cancer perception subscales and HPLP-II score.

Predictor	B (Unstandardized)	SE	t	*p*	95% CI
Perceived Knowledge	0.0592	0.0141	4.21	<0.001	0.0316 to 0.0867
Perceived Treatment Belief	0.1204	0.0215	5.59	<0.001	0.0782 to 0.1627
Perceived Need for Health Check	−0.2587	0.0173	−14.91	<0.001	−0.2927 to −0.2247
Perceived Stigma	0.0578	0.0179	3.23	0.001	0.0226 to 0.0929
Perceived Fear	−0.0284	0.0131	−2.17	0.030	−0.0541 to −0.0027
Perceived Risk	−0.1296	0.0237	−5.46	<0.001	−0.1762 to −0.0830

Note: *n* = 1002; Residual df = 995. B denotes unstandardized coefficients. The model includes all six BCPS subscales but is not adjusted for sociodemographic characteristics. Overall model-fit statistics (including R^2^, adjusted R^2^, and F-statistic) and complete regression diagnostics remain unavailable from the original analysis files.

### 3.6. Associations Between Sociodemographic Characteristics, Breast Cancer Perception Subscales, and HPLP-II Score

A separate fully adjusted 14-predictor model as shown in Table 6, was also examined to account for sociodemographic characteristics (R^2^ = 0.429, adjusted R^2^ = 0.421). Higher HPLP-II scores were associated with perceived treatment belief (B = 0.0942, 95% CI 0.0603–0.1281, *p* < 0.001), perceived knowledge (B = 0.0415, 95% CI 0.0193–0.0637, *p* < 0.001), and perceived stigma (B = 0.0405, 95% CI 0.0124–0.0686, *p* = 0.005). Perceived need for a health check had the largest inverse coefficient (B = −0.2091, 95% CI −0.2374 to −0.1808, *p* < 0.001), and perceived risk was also inversely associated (B = −0.0972, 95% CI −0.1333 to −0.0611, *p* < 0.001). Fear was not statistically significant (B = −0.0185, 95% CI −0.0389 to 0.0019, *p* = 0.075).

**Table 6:** Fully adjusted multivariable linear regression examining associations between sociodemographic characteristics, breast cancer perception subscales, and HPLP-II score.

Variable/Predictor	B (Unstandardized)	SE	t	*p*	95% CI	VIF
Age (continuous)	−0.0017	0.0011	−1.55	0.121	−0.0039 to 0.0005	1.44
Married (ref. Unmarried)	0.3392	0.0301	11.27	<0.001	0.2801 to 0.3983	1.57
High Schooling (ref. Primary)	−0.0041	0.0352	−0.12	0.907	−0.0732 to 0.0650	1.31
Secondary Schooling (ref. Primary)	−0.3785	0.0445	−8.51	<0.001	−0.4658 to −0.2912	1.63
University (ref. Primary)	−0.0498	0.0324	−1.54	0.124	−0.1133 to 0.0137	1.74
Public Official (ref. Not working)	0.1004	0.0308	3.26	0.001	0.0400 to 0.1608	1.50
Self-employed (ref. Not working)	0.0612	0.1192	0.51	0.608	−0.1727 to 0.2951	1.12
Family History: Yes (ref. No)	0.0305	0.0302	1.01	0.313	−0.0288 to 0.0898	1.09
Perceived Knowledge	0.0415	0.0113	3.67	<0.001	0.0193 to 0.0637	1.39
Perceived Treatment Belief	0.0942	0.0173	5.45	<0.001	0.0603 to 0.1281	1.43
Perceived Need for Health Check	−0.2091	0.0144	−14.52	<0.001	−0.2374 to −0.1808	1.47
Perceived Stigma	0.0405	0.0143	2.83	0.005	0.0124 to 0.0686	1.34
Perceived Fear	−0.0185	0.0104	−1.78	0.075	−0.0389 to 0.0019	1.41
Perceived Risk	−0.0972	0.0184	−5.28	<0.001	−0.1333 to −0.0611	1.42
Constant	2.2210	0.0725	30.63	<0.001	2.0788 to 2.3632	NA

Note: *n* = 1002; Residual df = 987; R^2^ = 0.429; adjusted R^2^ = 0.421; F(14, 987) = 52.97, *p* < 0.001; residual standard error = 0.349. B denotes unstandardized coefficients. The model includes all sociodemographic characteristics from Table 1 and all six BCPS subscales. VIF range = 1.09–1.74. NA indicates not applicable.

## 4. Discussion

This cross-sectional online survey described breast cancer perceptions, health-promoting lifestyle behaviours, and lifetime examination and mammography practices among 1002 women in Abha. The principal findings were as follows: (a) perceived knowledge, treatment beliefs, perceived risk, and fear had higher mean scores than perceived need for health checks and stigma; (b) spiritual growth was the highest-scoring HPLP-II domain and physical activity the lowest; (c) lifetime BSE (61.1%) was more commonly reported than CBE (22.5%) and mammography (12.8%); and (d) several BCPS domains were associated with HPLP-II scores in exploratory and fully adjusted models. Because the sample was recruited by convenience and screening was not modelled against perception, the findings do not support population-representative, causal, or awareness-to-screening conclusions.

### 4.1. Breast Cancer Perception and Health-Promoting Lifestyle Profile

Perceived knowledge had the highest mean score among the perception subscales (3.64, SD 1.05), while perceived need for health checks had the lowest (2.78, SD 1.23). Treatment belief, perceived risk, and fear also had relatively high scores. This pattern, in which women feel knowledgeable about breast cancer yet do not feel a strong need for routine health checks, is consistent with the awareness-practice gap reported in Saudi studies. Alqahtani et al. [7], studying women in the same Aseer region, found that more than 90% of participants had adequate knowledge of BSE, yet only 6.37% reported always performing it; similar gaps have been reported in Riyadh and Arar [5,6]. The relatively high fear and perceived risk scores should not be equated with screening behaviour. Fear of discovering cancer was reported in 85.7% of the studies included in a Saudi systematic review, making it the most frequently reported barrier across those studies [9].

The overall HPLP-II mean was 2.63 (SD 0.46), with spiritual growth highest (2.96) and physical activity lowest (2.23), a pattern also reported in previous Saudi studies using the instrument. Among Saudi medical students, Alzahrani et al. [13] found spiritual growth to be the highest and physical activity the lowest HPLP-II subscale, and a similar pattern was reported among Saudi postmenopausal women [12]. Spiritual growth was also the highest-scoring domain among Saudi medical students in a separate analysis, although health responsibility rather than physical activity scored lowest in that study [19]. Among Saudi undergraduates, spiritual growth was likewise highest and physical activity lowest [20]. These comparisons are descriptive and do not establish that the pattern is stable across all Saudi women.

The low physical activity score is relevant to breast cancer prevention because the World Cancer Research Fund/American Institute for Cancer Research reports strong evidence that being physically active decreases the risk of postmenopausal breast cancer, whereas greater adult body fatness and weight gain increase that risk [11]. Nevertheless, the cross-sectional findings cannot determine whether risk perception influences activity, activity influences perception, or both reflect other factors.

### 4.2. Prevalence of Breast Cancer Screening Practices

In this sample, 61.1% reported lifetime BSE, 22.5% reported CBE, and 12.8% reported mammography; among women aged ≥40 years, 30.2% reported lifetime mammography. These are lifetime proportions, not screening coverage within recommended intervals. In the Saudi Health Interview Survey, 92% of women aged 50 years or older reported never having had a mammogram, and 89% had not received a CBE in the previous year [4]. A separate reanalysis of women aged ≥35 years from the same survey reported that 6.5% had ever undergone mammography [21]. A more recent multiregional quota-based survey reported 30.5% overall and 52.2% among women aged ≥40 years [22]. Our figures add clinical context but do not show improvement or decline. Differences in age composition, eligibility, sampling, geography, screening indication, and recall period make direct comparisons over time inappropriate.

BSE was more commonly reported in this sample (61.1%) than in earlier Saudi studies reporting ever-BSE rates of 38.5% in Madinah [23] and 41.6% in Riyadh [5], whereas lifetime mammography was lower than the approximately 40% reported in the Aseer region [7]. These differences may reflect variation in age composition, sampling, setting, and measurement definitions rather than true temporal change. BSE is a self-examination practice and should not be interpreted as equivalent to mammography-based population screening. Free service provision [4] does not by itself establish why uptake differs, and our data did not test psychological, perceptual, or organizational barriers to screening.

### 4.3. Sociodemographic Correlates of HPLP-II Score

In the multivariable model, marital status was by far the strongest sociodemographic correlate of health-promoting lifestyle: married women scored 0.51 points higher than unmarried women (B = 0.51, *p* < 0.001). Being a public official was also positively associated (B = 0.15, *p* < 0.001), while age showed a very small inverse association (B = −0.003 per year, *p* = 0.038), and family history of breast cancer was not associated with lifestyle score (*p* = 0.212). Marital status has also been identified as a facilitator of mammography uptake in Saudi studies, and a systematic review classified male family-member support as an interpersonal facilitator [24]. This screening literature does not explain the lifestyle association observed here; residual confounding and selection effects remain possible.

The education pattern was not monotonic: relative to primary education, secondary schooling was associated with lower HPLP-II scores (B = −0.53, *p* < 0.001), university education showed a smaller inverse association (B = −0.09, *p* = 0.031), and high schooling was not statistically significant (*p* = 0.864). These findings do not show that lower education promotes healthier lifestyles. The primary-education reference group included only 32 participants, and self-employment estimates were based on six participants, making these estimates potentially unstable. In the sensitivity analysis excluding these groups, coefficients for marital status, public-official status, and the BCPS domains changed by no more than 4%, although education contrasts using primary education as the reference could not be compared directly. Unequal category sizes, selection effects, and residual confounding remain possible explanations.

### 4.4. Association of Breast Cancer Perception Predictors with HPLP-II Scores

In the exploratory model, all six perception subscales were associated with a health-promoting lifestyle. Treatment belief, knowledge, and stigma were positively associated, whereas need for health checks, risk, and fear were inversely associated. Treatment belief had the strongest positive coefficient, and the need for health checks had the strongest inverse coefficient. After full sociodemographic adjustment, the BCPS coefficients were attenuated, and fear was no longer statistically significant (B = −0.019, *p* = 0.075). The directions of the other associations remained unchanged. These differences may be attributed to the different sets of covariates included in the exploratory BCPS-only model and the fully adjusted model. 

Read through the lens of the Health Belief Model, which underpins the BCPS [10,25], the positive coefficient for treatment belief is compatible with the perceived-benefits construct. However, the cross-sectional association does not show that treatment beliefs lead women to engage in health-protective behaviour. The inverse coefficient for perceived need for health checks also warrants cautious interpretation because item wording, reverse coding, confounding, and the general lifestyle outcome may contribute to the observed pattern.

The inverse associations of fear and perceived risk with lifestyle differ from a simple expectation that perceived threat necessarily motivates protective action. Previous Saudi literature has identified fear as a barrier to screening [9], and a systematic review reported that anxiety about screening can deter participation whereas worry about diagnosis can sometimes encourage it [26]. Our results do not establish that fear leads to avoidance. The positive stigma coefficient is also counterintuitive and may reflect confounding, shared variance, or suppression rather than a facilitating role of stigma.

### 4.5. Implications of the Findings

The findings suggest several areas for further investigation rather than establishing intervention effects. First, awareness campaigns could be evaluated with behavioural components addressing fear of diagnosis [9], beliefs that screening is necessary only when symptoms occur [27], and concerns about the screening procedure [26]. However, our data did not test whether these factors mediate screening participation or whether changing them improves uptake.

Second, systematic referral of age-eligible women during primary care visits warrants evaluation. Physician advice was reported as a screening motivator by 41.8% of respondents in a national survey [28], and lack of provider recommendation was identified as a barrier in a systematic review [24]. Our study did not measure referral, provider recommendation, or screening indication.

Third, outreach research should consider whether online recruitment and unequal access contribute to differences across marital, educational, and employment groups. The observed associations should not be used to label any group as disadvantaged without accounting for selection and residual confounding.

Fourth, family-engaged screening promotion warrants evaluation: a systematic review identified male family-member support as an interpersonal facilitator [24], and research in Abha documented limited breast cancer knowledge among male partners [29]. Culturally acceptable physical activity programmes are a separate area for prevention research, given the WCRF/AICR evidence on physical activity and postmenopausal breast cancer risk [11]. These strategies require prospective evaluation; the present cross-sectional associations cannot establish their likely effectiveness.

### 4.6. Limitations

Several limitations should be considered when interpreting these findings. First, the cross-sectional design precludes causal inference; the observed associations between perception and lifestyle may be bidirectional, and the direction of effect cannot be determined.

Second, the convenience sample recruited online through social media and instant messaging is not representative of all women in Abha or the Aseer region. Internet-based recruitment may favour younger, more educated, and more digitally engaged women. Open-link distribution prevented calculation of a participation or response rate, and self-reported nationality, residence, and eligibility could not be independently verified.

Third, all measures were self-reported, exposing the study to social desirability, recall, and reporting bias. Examination and mammography items used lifetime ever/never responses without dates or clinical indication, so recommended-interval adherence and screening-eligible coverage could not be evaluated. The overall mammography proportion includes women outside routine screening age groups and should not be compared directly with age-restricted estimates. BSE is not equivalent to mammography-based population screening.

Fourth, the translation procedures and present-sample internal-consistency estimates do not establish the full construct validity of the Arabic BCPS and HPLP-II in this population. The original Arabic validation of the earlier HPLP likewise does not establish psychometric validity of the complete 52-item HPLP-II. Fifth, Table 5 lacked sociodemographic adjustment, and its model-fit statistics and complete diagnostics were unavailable. In contrast, Table 6 was fully adjusted, and its model fit and regression diagnostics were verified. No multiplicity adjustment was applied, so nominal *p*-values should be interpreted cautiously.

Finally, estimates involving primary education (*n* = 32) and self-employment (*n* = 6) may be unstable. The sensitivity analysis supported the stability of the core marital-status, public-official, and BCPS coefficients but did not improve the precision of the small-category estimates or permit direct comparison of education contrasts after primary education was excluded. Residual confounding by unmeasured factors, including income, parity, previous exposure to awareness campaigns, and access to female providers, cannot be excluded.

## 5. Conclusions

In this online convenience sample of women in Abha, Saudi Arabia, lifetime mammography and CBE were reported less frequently than BSE, and physical activity was the lowest-scoring HPLP-II domain. BCPS subscales showed cross-sectional associations of varying magnitude with HPLP-II scores in exploratory and fully adjusted models, but these findings may remain confounded, their practical importance is uncertain, and they do not establish causal pathways. The data also do not test whether awareness or perception predicts screening. Future studies should use probability-based recruitment where feasible, validated Arabic instruments, age- and indication-specific screening measures, and longitudinal designs before evaluating targeted interventions.

## Figures and Tables

**Table 1:** Participant demographic and study-sample characteristics (*n* = 1002).

Characteristic	Category or Summary	*n*	%
Total sample	All participants	1002	100.0
Age	Aged ≥18 years (eligibility criterion)	1002	100.0
Age summary	Mean ± SD (observed range): 32.4 ± 9.8 years (18–68)	NA	NA
Age group	18–29 years	465	46.4
	30–39 years	312	31.1
	40–49 years	148	14.8
	≥50 years	77	7.7
Sex	Women (self-identified)	1002	100.0
Nationality	Saudi (self-reported)	1002	100.0
Residence	Abha (self-reported)	1002	100.0
Previous breast cancer diagnosis	No	1002	100.0
Marital status	Married	584	58.3
	Unmarried (single/divorced/widowed)	418	41.7
Educational attainment	Primary	32	3.2
	High schooling	84	8.4
	Secondary schooling	215	21.5
	University (bachelor’s/postgraduate)	671	67.0
Employment	Not working (students/homemakers/unemployed)	428	42.7
	Public official (government/education/healthcare)	568	56.7
	Self-employed	6	0.6
Family history of breast cancer	Yes	186	18.6
	No	816	81.4

Note: Percentages use *n* = 1002 and were calculated from the displayed counts; because of independent rounding, the education percentages sum to 100.1%. NA indicates that the summary is not a count or percentage. The unmarried category combines single, divorced, and widowed participants; the not-working category combines students, homemakers, and unemployed participants; high schooling denotes completion of Grades 10–12 in the general curriculum; secondary schooling denotes vocational or technical post-secondary certification; and the university category includes bachelor’s and postgraduate education.

**Table 2:** Descriptive statistics for breast cancer perception scores and HPLP-II subscales.

Variable	Mean	SD	Minimum	Maximum
Perceived Knowledge	3.64	1.05	1.75	5.00
Perceived Treatment Belief	3.43	0.77	1.80	4.60
Perceived Need for Health Check	2.78	1.23	1.00	5.00
Perceived Stigma	2.89	1.14	1.50	5.00
Perceived Fear	3.25	1.30	1.00	5.00
Perceived Risk	3.40	0.89	2.00	5.00
Health Responsibility (HR)	2.53	0.65	1.67	4.00
Physical Activity (PA)	2.23	0.60	1.50	4.00
Nutrition (NUT)	2.67	0.43	2.00	4.00
Spiritual Growth (SG)	2.96	0.50	2.11	4.00
Interpersonal Relations (IR)	2.65	0.50	2.00	4.00
Stress Management (SM)	2.66	0.60	1.88	4.00
HPLP-II Score	2.63	0.46	2.00	4.00

Note: Final scores were calculated as unweighted arithmetic means across the exact item counts for all 1002 complete cases, without imputation. The minimum values were 1.67 for health responsibility, 2.11 for spiritual growth, and 1.88 for stress management. The minimum of 2.00 observed for the total score, nutrition, and interpersonal relations indicates restricted lower-tail variation but does not, by itself, establish a floor effect or truncation. Item-level distributional checks would be needed to distinguish response patterns from data-handling artefacts.

**Table 3:** Age-stratified lifetime self-reported breast examination and mammography history (*n* = 1002).

Stratum/Subgroup	*n*	BSE *n* (%) [95% CI]	CBE *n* (%) [95% CI]	Mammography *n* (%) [95% CI]
Full sample	1002	612 (61.1) [58.1–64.1]	225 (22.5) [19.9–25.0]	128 (12.8) [10.7–14.8]
Age 18–29 years	465	271 (58.3) [53.8–62.8]	62 (13.3) [10.2–16.4]	18 (3.9) [2.1–5.6]
Age 30–39 years	312	204 (65.4) [60.1–70.7]	89 (28.5) [23.5–33.5]	42 (13.5) [9.7–17.2]
Age 40–49 years	148	98 (66.2) [58.6–73.8]	51 (34.5) [26.8–42.1]	45 (30.4) [23.0–37.8]
Age ≥50 years	77	39 (50.6) [39.5–61.8]	23 (29.9) [19.6–40.1]	23 (29.9) [19.6–40.1]
Age ≥40 years subgroup	225	137 (60.9) [54.5–67.3]	74 (32.9) [26.8–39.0]	68 (30.2) [24.2–36.2]

Note: Values are *n* (%) with descriptive 95% confidence intervals calculated using the binomial normal approximation. The ≥40-year subgroup comprises 148 participants aged 40–49 years and 77 aged ≥50 years. Age-based subgrouping does not capture individual risk-based eligibility, and lifetime history should not be equated with recommended-interval or guideline-concordant screening coverage.

## Data Availability

The data presented in this study are available upon request from the corresponding author.
